# The Immune Adaptor ADAP Regulates Reciprocal TGF-β1-Integrin Crosstalk to Protect from Influenza Virus Infection

**DOI:** 10.1371/journal.ppat.1004824

**Published:** 2015-04-24

**Authors:** Chunyang Li, Shaozhuo Jiao, Guojun Wang, Yunzhen Gao, Chang Liu, Xijun He, Chi Zhang, Jun Xiao, Weiyun Li, Guoquan Zhang, Bin Wei, Hualan Chen, Hongyan Wang

**Affiliations:** 1 Key Laboratory of Systems Biology, Innovation Center for Cell Signaling Network, Institute of Biochemistry and Cell Biology, Shanghai Institutes for Biological Sciences, Chinese Academy of Sciences, Shanghai, China; 2 State Key Laboratory of Veterinary Biotechnology, Harbin Veterinary Research Institute, Chinese Academy of Agricultural Science, Harbin, China; 3 State Key Laboratory of Virology, Wuhan Institute of Virology, Chinese Academy of Sciences, Wuhan, China; Johns Hopkins University - Bloomberg School of Public Health, UNITED STATES

## Abstract

Highly pathogenic avian influenza virus (HPAI, such as H5N1) infection causes severe cytokine storm and fatal respiratory immunopathogenesis in human and animal. Although TGF-β1 and the integrin CD103 in CD8^+^ T cells play protective roles in H5N1 virus infection, it is not fully understood which key signaling proteins control the TGF-β1-integrin crosstalk in CD8^+^ T cells to protect from H5N1 virus infection. This study showed that ADAP (Adhesion and Degranulation-promoting Adapter Protein) formed a complex with TRAF6 and TAK1 in CD8^+^ T cells, and activated SMAD3 to increase autocrine TGF-β1 production. Further, TGF-β1 induced CD103 expression via an ADAP-, TRAF6- and SMAD3-dependent manner. In response to influenza virus infection (i.e. H5N1 or H1N1), lung infiltrating ADAP^-/-^ CD8^+^ T cells significantly reduced the expression levels of TGF-β1, CD103 and VLA-1. ADAP^-/-^ mice as well as Rag1^-/-^ mice receiving ADAP^-/-^ T cells enhanced mortality with significant higher levels of inflammatory cytokines and chemokines in lungs. Together, we have demonstrated that ADAP regulates the positive feedback loop of TGF-β1 production and TGF-β1-induced CD103 expression in CD8^+^ T cells via the TβRI-TRAF6-TAK1-SMAD3 pathway and protects from influenza virus infection. It is critical to further explore whether the SNP polymorphisms located in human *ADAP* gene are associated with disease susceptibility in response to influenza virus infection.

## Author Summary

Infection of avian influenza virus, especially the highly pathogenic strain H5N1, is a serious threat to public health worldwide, which causes severe fatal respiratory disease and excessive levels of inflammation. It has been reported that both transforming growth factor-beta 1 (TGF-β1) and the integrin CD103 induced by TGF-β1 play protective roles in influenza virus infections. We aimed to find which protein regulates the TGF-β1-integrin cross-talk to protect against H5N1 virus infection. This study provides the first evidence that the intracellular signaling protein ADAP (adhesion and degranulation-promoting adapter protein) up-regulates TGF-β1 production and TGF-β1 induced CD103 expression in CD8^+^ T cells via the TβRI-TRAF6-TAK1-SMAD3 pathway. Importantly, in response to H5N1 and H1N1 virus infection, ADAP deficiency decreases TGF-β1 production and CD103 expression in lung infiltrating CD8^+^ T cells with the enhanced mortality in mice. Since various SNPs or mutations in key molecules of TGF-β1 pathway, including polymorphisms located in *ADAP/FYB* gene, are associated with inflammatory diseases, future work should investigate whether these SNPs or mutations enhance disease susceptibility or clinical manifestations in response to acute influenza virus infection.

## Introduction

H5N1 influenza viruses are highly pathogenic avian influenza (HPAI) virus, which also infect humans and cause fatal human respiratory diseases [1, 2]. Numerous animal or clinical studies have indicated that virus-induced cytokine dysregulation is one central reason for H5N1 pathogenesis and disease severity [2–4]. Compared with the influenza virus subtype H1N1, H5N1-infected patients showed unusually high serum levels of chemokines and inflammatory cytokines. And, H5N1-infected patients who died had higher serum levels of these mediators than those who survived [1, 2]. Mouse studies also suggest that sustained induction of the inflammatory response after H5N1 virus infection is correlated with the ability of H5N1 virus to disseminate to extrapulmonary organs [5]. Although the appearance of cytokine storm (also termed hypercytokinemia) is one of the most important features of H5N1 and H1N1 immunopathogenesis, mouse models deficient of cytokines IL-6 or MIP-1α show comparable mortality as influenza-challenged wild type controls [4, 6]. This might be due to the redundancy between cytokines or chemokines. Therefore, identification of other strategies to prevent cytokine storm or damage of respiratory tract is crucial and should shed new light on effective clinical anti-H5N1 or H1N1 treatment.

Transforming growth factor β1 (TGF-β1) is the predominant member of the TGF-β isoforms in the immune system, and controls initiation and resolution of inflammation during multiple pathogenic progress, including influenza virus infection [7, 8]. TGF-β is secreted as an inactive precursor, which cannot bind to its receptor in its latent form (i.e. LTGF-β) [9]. The neuraminidase glycoprotein (NA) of most influenza strains including H1N1 could activate latent TGF-β (LTGF-β), while H5N1 cannot activate latent TGF-β [10, 11]. In agreement with the protective role of TGF-β during influenza virus infection, exogenous TGF-β delays mortality in H5N1-infected mice, and depletion of TGF-β increases mortality of H1N1 infection [10]. We aimed to dissect new mechanism of TGF-β production in host cells to ameliorate H5N1 or H1N1 virus infection.

Integrins have been identified to play critical roles in the regulation of TGF-β1 activation. Furthermore, once activated TGF-β1 binds to its heterodimeric receptor complex TβRI/II to induce SMAD-dependent or independent pathways, expression of multiple integrins is enhanced, including CD103 (also termed αEβ7) and VLA-1 (very late antigen-1, also termed α1β1) [12]. CD103 is preferentially expressed on CD8^+^ T cells specific for influenza but not for EBV or CMV, and maintains CD8^+^ T cells in lungs by binding to its ligand epithelial cadherin (E-Cadherin), which enhances immune surveillance to clear influenza infection [13, 14]. Despite of this, it remains to be elucidated which cytoplasmic effector(s) plays an indispensable role in the control of reciprocal TGF-β1-integrin crosstalk.

We and others have demonstrated that upon infection or chemokine stimulation, T cells use the immune adaptor protein ADAP (adhesion and degranulation-promoting adapter protein, also known as Fyn-binding protein [Fyb] or SLP-76 associated protein of 130 kD [SLAP-130]) to increase β2 integrin activation and adhesion [15–17]. However, it remains unclear whether ADAP regulates the expression of TGF-β1, CD103 and VLA-1 or controls the reciprocal TGF-β1-integrin crosstalk during influenza infection. In this study, we provided the first evidence that ADAP was indispensable for autocrine TGF-β1 production and CD103 expression via the TβR-TRAF6-ADAP-TAK1-SMAD3 pathway. Importantly, ADAP knock-out (KO) mice as well as Rag1 KO mice receiving ADAP KO T cells enhanced mortality in response to H5N1 or H1N1 infection, which displayed severe cytokine storm and lung damages with reduced levels of TGF-β1, CD103 and VLA-1 in CD8^+^ T cells.

## Results

### ADAP regulates TGF-β1 production in T cells via an autocrine manner

**Fig 1:**
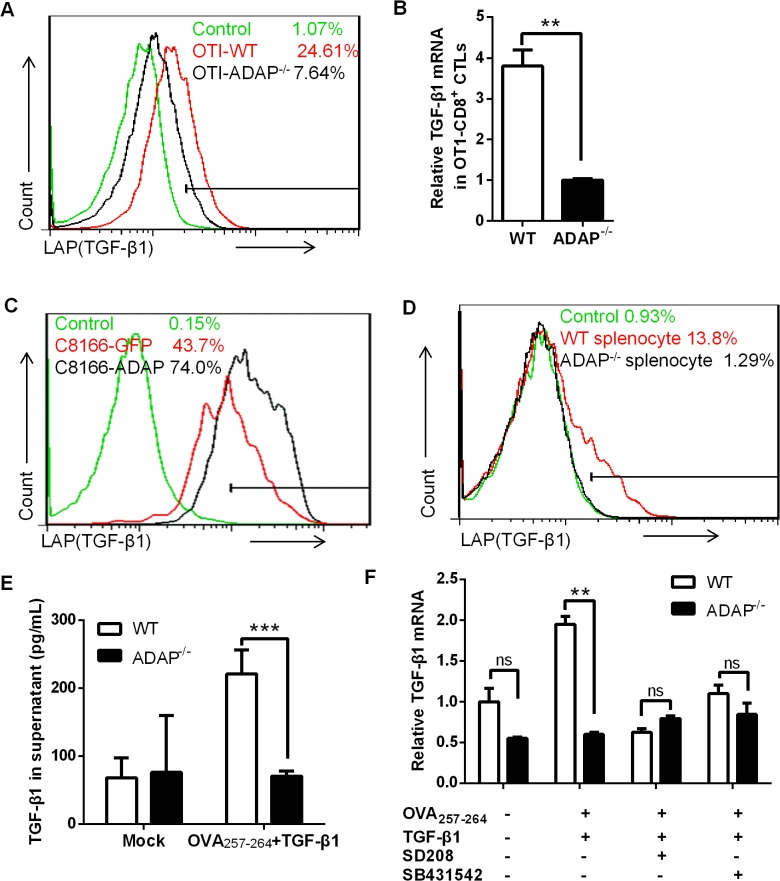
ADAP regulates TGF-β1 production in T cells via an autocrine manner. (A and B) Surface staining of LAP (TGF-β1) and the relative mRNA levels of TGF-β1 in CD8^+^ CTLs from wild type or ADAP^-/-^ OT1 Tg mice, which were generated with 10nM OVA_257-264_-peptide stimulation for 3 days. Data are representative of three independent experiments. (C) Human C8166 T cells overexpressing GFP or ADAP were stimulated with 2ug/mL plate bound anti-CD3/CD28 antibodies, followed by surface staining of LAP (TGF-β1). Data are representative of two independent experiments. (D) Wild type or ADAP^-/-^ splenocytes were stimulated with 2ug/mL plate bound anti-CD3/CD28 antibodies, followed by surface staining of CD8 and LAP (TGF-β1). Data are representative of three independent experiments. (E) After stimulation with 5ng/mL TGF-β1 and 10nM OVA_257-264_-peptide for 6hrs, Wild type or ADAP^-/-^ splenocytes were washed extensively and then incubated for further 18hr. Supernatants were collected to detect total TGF-β with ELISA. (F) In the presence or absence of the inhibitors SD208 and SB431542 (10uM respectively), the mRNA levels of TGF-β1 were examined in wild type or ADAP^-/-^ splenocytes after stimulated with 10nM OVA_257-264_-peptide with or without 5ng/mL exogenous TGF-β1. Data are representative of three independent experiments.

ADAP is an intracellular hematopoietic-specific adaptor protein that is constitutively and mainly expressed in T lymphocytes. Multiple studies have demonstrated that ADAP plays a crucial role in enhancing β2 integrin activation in response to antigen or chemokine stimulation [15, 16, 18, 19]. Since TGF-β1 and integrins have close relationship and function on each other, we asked whether ADAP regulated the crosstalk between integrins and TGF-β1. We first examined whether ADAP regulated TGF-β1 production in CD8^+^ cytotoxic T lymphocytes (CTLs). ADAP^-/-^ mice were crossed with OT1 TCR transgenic (OT1 Tg) mice to obtain CD8^+^ Tg cells, which express specific TCR for recognizing OVA_257-264_ peptide. Splenocytes from wild type or ADAP^-/-^ OT1 mice were incubated with OVA_257-264_ peptide for 7 days to generate OVA_257-264_-specific CD8^+^ CTLs. LAP (TGF-β1) is TGF-β1 precursor containing LAP (latency-associated peptide) and anti-LAP (TGF-β1) antibody is used to detect the latent form of TGF-β1. Compared to that of wild type cells, we observed that OVA-stimulated ADAP^-/-^ CD8^+^ CTLs significantly reduced TGF-β1 production at precursor levels by anti-LAP (TGF-β1) staining (7.64% vs. 24.61%) or at mRNA levels by RT-PCR analysis (Fig 1A and 1B). Next, ADAP was overexpressed in human C8166 T cell line, followed by stimulation with anti-CD3/CD28 antibodies. ADAP overexpression enhanced LAP (TGF-β1) surface expression compared to the GFP controls (Fig 1C, 74% vs. 43.7%). In agreement with this observation, wild type splenocytes enhanced LAP (TGF-β1) production after anti-CD3/CD28 stimulation, while ADAP^-/-^ splenocytes failed to increase LAP (TGF-β1) production (1.29% vs. 13.8%, Fig 1D). Next, we measured the mature/active form and the inactive form of TGF-β1 by ELISA assay in wild type and ADAP^-/-^ CD8^+^ CTLs after stimulation with OVA_257-264_ peptide and exogenous TGF-β1. According to the manufactory’s instruction, the mouse ELISA kit recognizes the mature/active form of TGF-β1. After samples received acid treatment and neutralization to remove LAP from TGF-β1 precursor, both active and inactive TGF-β1 in the supernatants could be detected by ELISA assay. We found that in response to OVA_257-264_ peptide and exogenous TGF-β1 stimulation, ADAP deficient CD8^+^ CTLs indeed decreased both active and inactive TGF-β1 concentrations (Fig 1E).

It has been demonstrated that TGF-β1 production is regulated by an auto-feedback loop via TGF-β receptor I/II (TβRI/II) [20, 21]. TGF-β1 binds to a type II receptor, which recruits and phosphorylates the type I receptor TβRI. We observed that treatment with exogenous TGF-β1 enhanced the mRNA levels of TGF-β1 in OVA_257-264_-stimulated wild-type CD8^+^ OT1 Tg cells, while ADAP^-/-^ CD8^+^ OT1 Tg cells did not show this enhancement (Fig 1E). Next, the TGF-β receptor I kinase inhibitor SD208 or SB431542 was used to inhibit TGF-β signaling. In the presence of SD208 or SB431542, exogenous TGF-β1 failed to effectively induce TGF-β1 production in wild type cells, which was reduced to similar levels as that in ADAP^-/-^ OT1 Tg cells (Fig 1F). Together, we suggest ADAP as a critical intracellular effector for autocrine TGF-β1 production in T cells, which is dependent on TβRI kinase activity.

### ADAP enhances TGF-β1/TβRI signaling via TRAF6-TAK1-SMAD3

**Fig 2:**
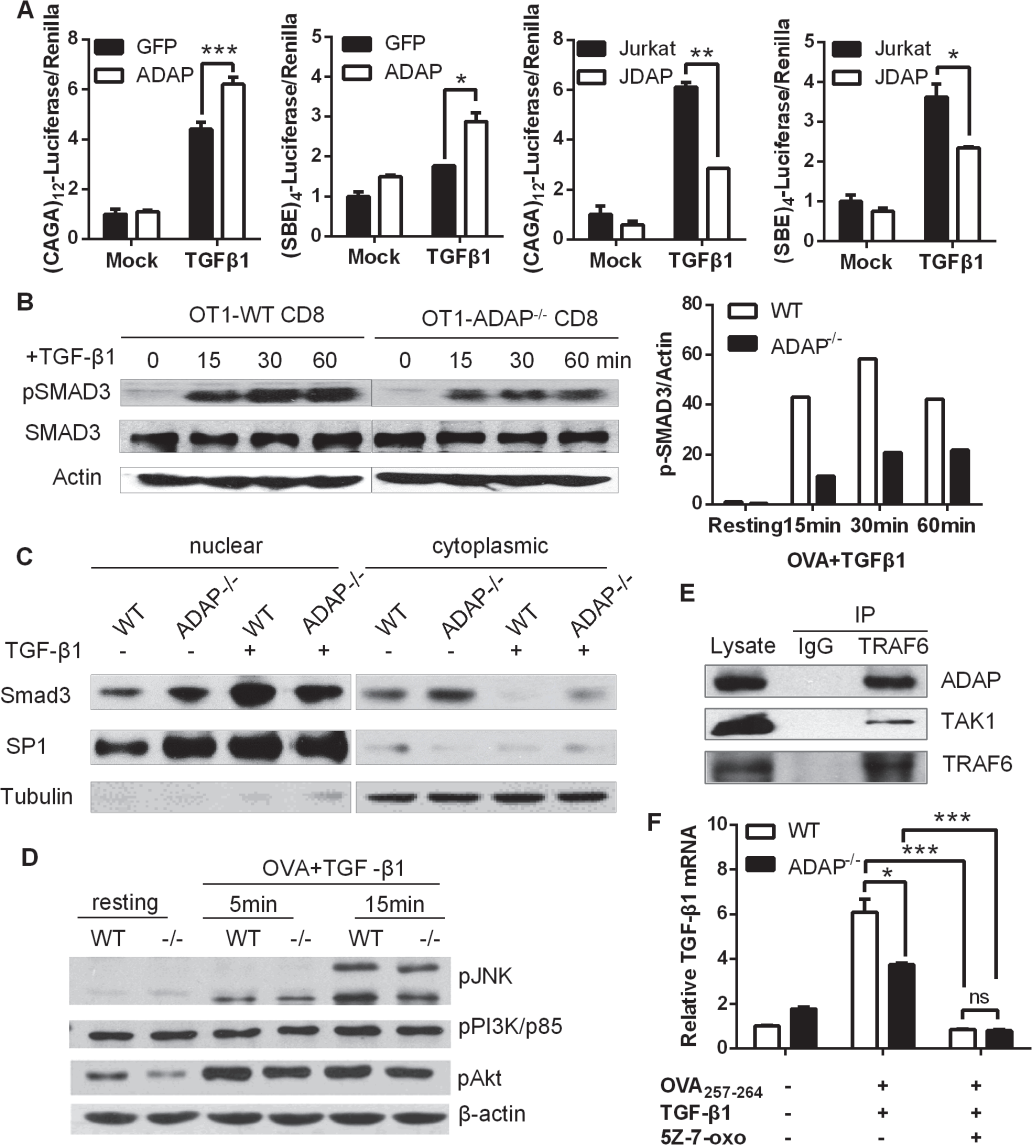
ADAP enhances TGF-β1/TβRI signaling via TRAF6-TAK1-SMAD3. (A) Wild type controls, ADAP-overexpressing Jurkat cells or ADAP deficient Jurkat cells (JDAP) were transfected with the (CAGA)_12_-luciferase and (SBE)_4_-luciferase reporters respectively. After exogenous TGF-β1 treatment (5ng/mL), the luciferase activity was assessed. Data are representative of two independent experiments. (B) Wild type or ADAP^-/-^ CD8^+^ OT1 CTLs were generated with 10nM OVA_257-264_ peptide stimulation, then treated with 5ng/mL exogenous TGF-β1 as the indicated time points. The levels of pSMAD3 (ser 423/425), total SMAD3 and β-actin were examined. The relative ratio between p-SMAD3 and β-actin was shown. Data are representative of two independent experiments. (C) WT and ADAP KO CD8^+^ CTLs were treated with 5ng/mL TGF-β1 for 30min, followed by preparation of cytoplasmic and nuclear extracts. Equal amounts of cytoplasmic and nuclear extracts were loaded for western blotting assay with antibodies against SMAD3. SP1 expression in nuclear extracts and tubulin expression in cytoplasmic extracts were used as controls. Data are representative of two independent experiments. (D) Wild type or ADAP^-/-^ CD8^+^ OT1 Tg cells were stimulated with 10nM OVA_257-264_ peptide and 5ng/mL exogenous TGF-β1 as the indicated time points. The levels of p-p38, p-JNK, p-p85 (Tyr458), p-AKT (Ser473) and β-actin were examined. Data are representative of two independent experiments. (E) Cell lysates from OT-I CD8^+^ CTLs were immunoprecipitated with anti-TRAF6 antibody, followed by immunoblotting with antibodies against ADAP, TAK1 and TRAF6. Data are representative of two independent experiments. (F)The mRNA levels of TGF-β1 were examined in CD8^+^ CTLs from wild type or ADAP^-/-^ OT1 Tg mice that were generated with 10nM OVA peptide and 5ng/mL exogenous TGF-β1 in the presence or absence of the 2uM TAK1 inhibitor 5Z-7-oxozeaenol. Data are representative of three independent experiments.

Upon TGF-β1 stimulation, TβRI recruits and phosphorylates R-SMADs (receptor-regulated Smads, i.e. SMAD2 or SMAD3). R-SMADs then bind the common SMAD4 and enter the nucleus as transcription factors to induce expression of various genes, including TGF-β1 itself. Given that ADAP was involved in TGF-β1/TβRI signaling for the induction of TGF-β1 (Fig 1), we asked whether ADAP regulated the TβRI/II-induced SMAD activity. Jurkat T cells were transfected with GFP control or ADAP-GFP together with the (CAGA)_12_-luciferase or (SBE)_4_-luciferase reporters respectively, which contain SMAD binding sequences [22, 23]. Exogenous TGF-β1 treatment increased the luciferase activity compared to untreated samples; and compared to the GFP control, ADAP overexpression further enhanced this by about 30% (Fig 2A, left two panels). Next, wild type or ADAP deficient Jurkat cells (i.e. termed JDAP) were transfected with the (CAGA)_12_-luciferase or (SBE)_4_-luciferase reporters respectively. In response to exogenous TGF-β1 treatment, wild type Jurkat cells increased the luciferase activity around two-folds compared to that in JDAP cells (Fig 2A, right two panels).

To further confirm whether ADAP regulated the transcriptional activity of R-SMAD, we measured the phosphorylation levels of R-SMAD. Compared to OVA_257-264_-stimulated wild type CD8^+^ OT1 Tg cells, ADAP^-/-^ CD8^+^ OT1 Tg cells induced lower levels of phosphorylated SMAD3 (p-SMAD3 ser 423/425) in response to exogenous TGF-β1 stimulation (Fig 2B, the relative p-SMAD3/actin ratio was shown in the right panel). ADAP deficiency did not affect the total amount of SMAD3 expression. In addition, nuclear and cytoplasmic extracts from wild type and ADAP-^/-^ CD8^+^ OT1 Tg cells were used to quantify the amount of nuclear localized SMAD3. TGF-β1 treatment induced SMAD3 nuclear translocation in wild type CD8^+^ CTLs, while ADAP deficiency decreased SMAD3 nuclear translocation compared to wild type cells (Fig 2C).

As an immune adaptor protein, ADAP does not have kinase or enzyme activity, but contains several domains (i.e. proline-rich domain, SH3 domain and multiple tyrosines) for recruitment of other signaling proteins. To explore how ADAP was involved in TGF-β1 signaling, we overexpressed ADAP with SMAD2 or SMAD3 in 293T cells respectively, but did not find direct interaction between ADAP and SMAD2 or SMAD3 (S1A Fig). We next examined whether ADAP regulated the non-canonical SMAD signaling that participate in TGF-β1 production. Previous studies have shown interesting feature of the PI3K/AKT pathway, the MAPKs (mitogen-activated protein kinases) including p38 and JNK (c-Jun N-terminal kinase) to modulate SMAD activity [24–28]. In response to TGF-β1 and OVA peptide stimulation, our representative data showed that wild type and ADAP^-/-^ CD8^+^ OT1 Tg cells displayed similar levels of p-p38, p-JNK, the PI3K subunit p-p85 (Tyr458) and p-AKT (Ser473) (Fig 2D, the relative ratio compared to β-actin was shown in S1B Fig).

TAK1 (TGF-β activated kinase 1) is another non-canonical SMAD signaling protein to regulate SMAD3 activation [29]. The TβRI-TRAF6 interaction was previously identified for TGF-β-induced autoubiquitylation of TRAF6 and subsequent activation of TAK1 [30]. Interestingly, when GFP-tagged ADAP was co-transfected with Flag-tagged TRAF6 or HA-tagged TAK1 respectively in 293T cells, we observed direct binding of ADAP to TRAF6 (S1C Fig, left panel). Although no direct interaction between ADAP and TAK1, co-transfection of TRAF6 could bring ADAP and TAK1 into the same complex (S1C Fig, right panel). We next examined whether endogenous ADAP interacted with TRAF6 in primary CD8^+^ CTLs. Anti-TRAF6 immunorecipitation was performed followed by immunoblotting with antibodies against ADAP and TAK1. We confirmed that endogenous TRAF6 indeed forms a complex with ADAP and TAK1 in primary CD8^+^ T cells (Fig 2E).

To test whether TAK1 played a central role together with ADAP in TGF-β1 production, wild type and ADAP^-/-^ CD8^+^ OT1 CTLs were stimulated with OVA peptide and exogenous TGF-β1 in the presence of the TAK1 inhibitor 5Z-7-oxozeaenol. The mRNA levels of TGF-β1 were profoundly decreased by 5Z-7-oxozeaenol treatment, and no difference was detected between wile type and ADAP^-/-^ CD8^+^ OT1 CTLs (Fig 2F). Together, these data suggest that ADAP forms a signaling module with TRAF6 and TAK1 to enhance TGF-β1-induced SMAD3 activation and TGF-β1 production via an autocrine manner.

### ADAP^-/-^ mice enhanced mortality with reduced TGF-1 production in response to H5N1 virus infection

**Fig 3:**
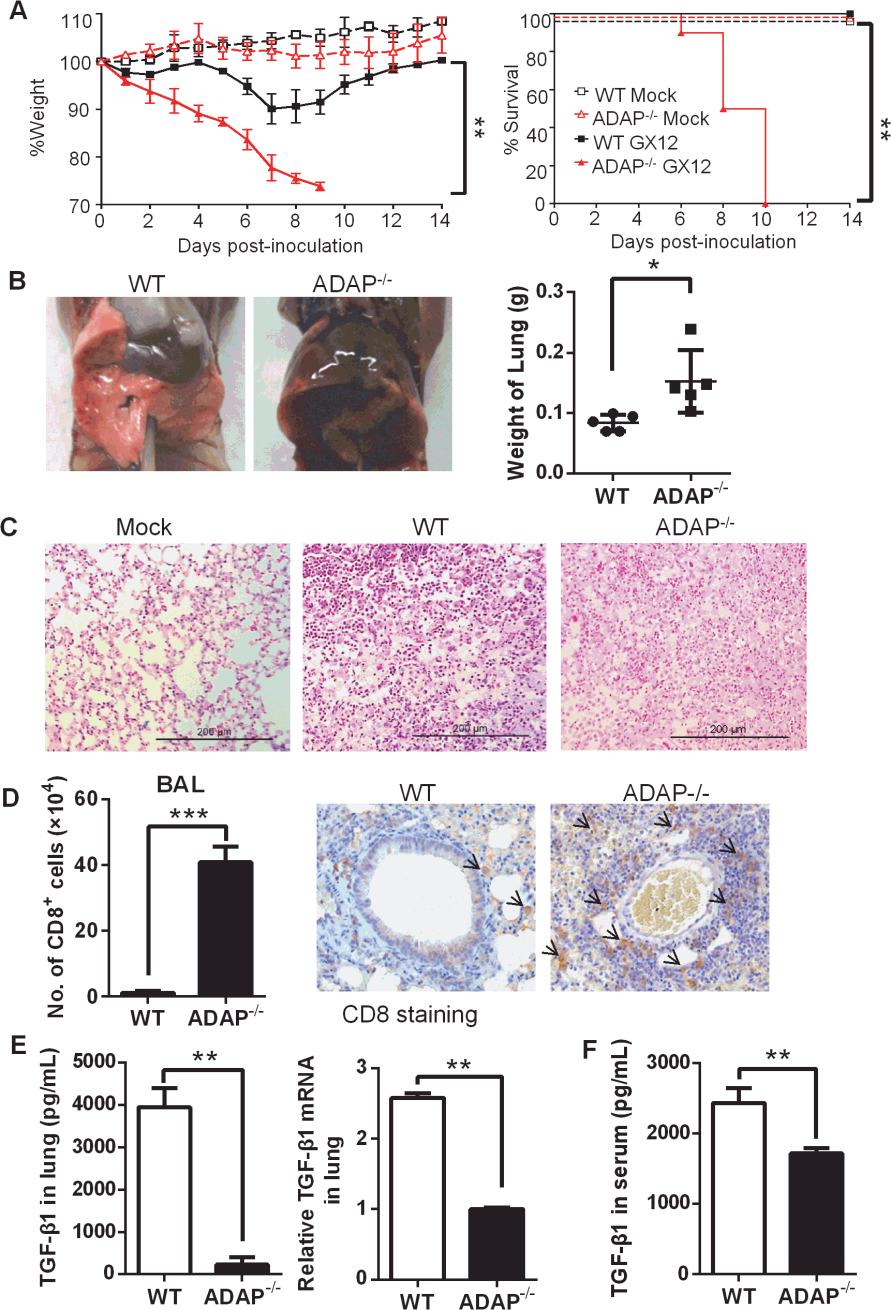
ADAP-/- mice enhanced mortality with reduced TGF-β1 production in response to H5N1 virus infection. Wild type and ADAP^-/-^ mice were infected with the H5N1 strain 10^6^ EID_50_ of GX/12 to examine their bodyweight changes & survival rates (A), lung features (B, C), number of lung infiltrating CD8^+^ T cells (D) and TGF-1 concentrations or mRNA levels in lungs (E) or serum TGF-1 concentrations (F) at day 10 post infection (Mock group: n = 5; GX/12 infected group: n = 10. Data are representative of at least two independent experiments.

Since TGF-β has been identified to play a protective role during the influenza H5N1 virus infection [10], we asked whether and how ADAP regulated H5N1 virus infection. Previously reports showed that ADAP deficient mice have normal T cell development in thymus and normal percentages or numbers of mature peripheral T cells [15–17]. Wild type and ADAP^-/-^ mice were challenged with the H5N1 strain A/DK/GuangXi/12/03 that was isolated from ducks in China (i.e. termed GX/12 in this paper) [31]. As reported earlier [31], the infected wild type mice started to recover from day 8 and remained survival. In contrast, the infected ADAP^-/-^ mice markedly lost bodyweight and 100% died around day 10 post infection (Fig 3A). In the PBS-treated mock control groups, ADAP^-/-^ mice and wild type mice showed normal lung structure without immune cell infiltration. At day 10 post infection, lungs from ADAP^-/-^ mice were swollen with increased weight compared to those from wild type mice (Fig 3B). Substantially, lung structure was disrupted with massive infiltration of immune cells in the H5N1-infected ADAP^-/-^ mice by H&E staining (Fig 3C). At day 10 post infection, the number of infiltrated CD8^+^ T cells into BAL (bronchoalveolar lavage) of lungs was significantly enhanced in the infected ADAP^-/-^ mice examined by FACS analysis or by IHC staining (Fig 3D). The number of infiltrating CD8^+^ T into BAL was also examined at different time points after infection. At day 3–4 post infection, detectable number of infiltrating CD8^+^ T cells was noticed in lungs of wild type or ADAP^-/-^ mice. At day 8–9, infiltration of wild type CD8^+^ T cells reached maximal levels, then declined with recovery of wild type mice from infection. By contrast, the number of infiltrating ADAP^-/-^ CD8^+^ T cells was continuously and substantially increased around day 10 post-infection and ADAP^-/-^ mice were unable to recover from infection (S2A Fig).

Despite of enhanced ADAP^-/-^ CD8^+^ T lymphocytes in the infected lungs, concentrations and the mRNA levels of TGF-β1 in lungs were severely decreased in H5N1-infected ADAP^-/-^ mice compared to those from wild type mice (Fig 3E). Serum TGF-β1 concentrations were also reduced from the infected ADAP^-/-^ mice (Fig 3F). Since TGF-β1 plays a key role in the development of Foxp3^+^CD4^+^ Treg cells and Treg cells also inhibit excessive inflammatory responses, we examined the percentage of Treg cells in H5N1-infected ADAP^-/-^ mice. ADAP deficiency did not affect the percentage of lung infiltrating Treg cells (S2B Fig). Previous studies suggested that ADAP deficient Treg cells mediated suppressive capacity at wild type levels when co-transferred with effector T cells into NOD/SCID mice to monitor recipients for diabetes; and ADAP deficient Treg cells inhibited TCR-stimulated effector T cell proliferation *in vitro* as that by wild type Treg cells [32]. Together, our findings show that after H5N1 virus infection, ADAP deficiency impairs TGF-β1 production with enhanced mortality and lung infiltrating CD8^+^ T cells.

Since TGF-β and ADAP have been demonstrated to regulate cell cycle and apoptosis [16–18, 33, 34], we examined whether T cell proliferation and apoptosis were affected in H5N1-infected ADAP^-/-^ mice. The BrdU incorporation assay confirmed no significant difference of CD8^+^ T cell proliferation in lungs, MLN or spleens between the infected ADAP^-/-^ and wild type mice (S3A, S3B and S3C Fig; left panels). Also, ADAP deficiency did not change CD8^+^ T cell apoptosis in lungs, MLN (mediastinal lymph-node), and spleens as examined by PI/Annexin V staining (S3A, S3B and S3C Fig; right panels). Interestingly, the number of CD8^+^ T cells was reduced in spleens of the infected ADAP^-/-^ mice (S3D Fig).

### Severe inflammation and lung damage of H5N1-infected ADAP^-/-^ mice

**Fig 4:**
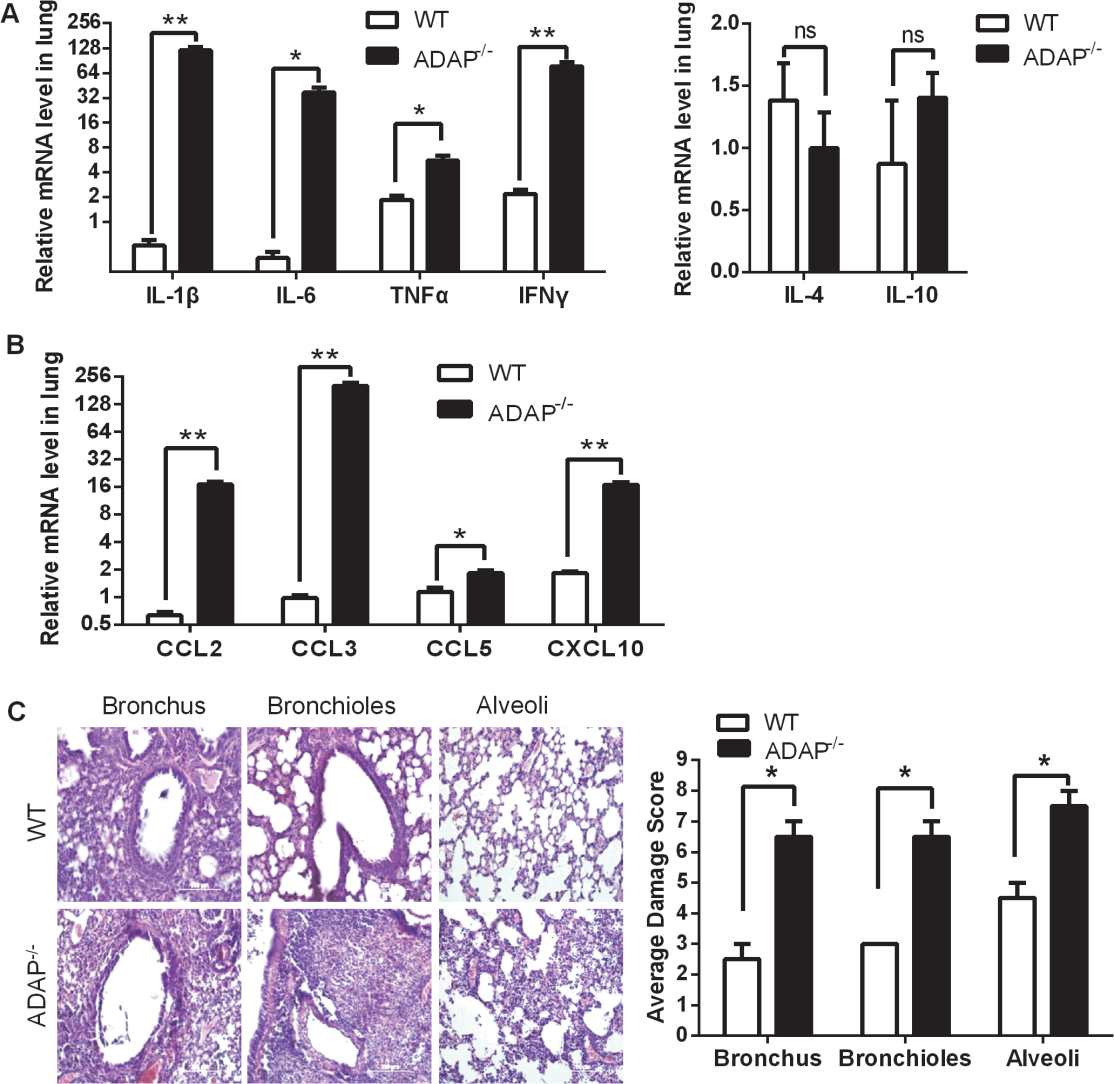
Severe inflammation and lung damage of H5N1-infected ADAP-/- mice. Wild type and ADAP^-/-^ mice were infected with the H5N1 virus and samples were collected when the bodyweight of the infected ADAP1^-/-^ mice were decreased by about 30% (i.e. at day 10) for the following analysis (n = 3): the mRNA levels of various cytokines (A) and chemokines (B); the average damage score of alveoli, bronchioles and bronchus of lungs by H&E staining (C). Data are representative of two independent experiments.

In agreement with the impaired TGF-β1 production in CD8^+^ T cells and ADAP^-/-^ lungs, we detected cytokine storm phenotype in H5N1-infected ADAP deficient mice. The relative mRNA levels of IL-1β, IL-6, TNFα and IFNγ were markedly increased in lungs from H5N1-infected ADAP^-/-^ mice with no significant changes of IL-4 (Fig 4A). Besides TGF-β1, IL-10 could also inhibit cytokine production. However, we found no significant difference of IL-10 mRNA levels in lungs between H5N1-infected wild type and ADAP^-/-^ mice (Fig 4A, right panel). Importantly, we also observed much higher levels of chemokines in lungs of H5N1-infected ADAP^-/-^ mice, including CCL2/MCP-1 (Monocyte Chemotactic Protein-1), CCL3/MIP-1α (Macrophage Inflammatory Protein-1α), CCL5 and CXCL10/IP-10 (Interferon-γ-inducible Protein) (Fig 4B). This might attract more immune cells to migrate from spleen to lung (S3D Fig) and enhance the number of lung infiltrating ADAP^-/-^ CD8^+^ T cells (Fig 3D).

Uncontrolled lung inflammation was account for the destructive structure of respiratory tract in H5N1-infected ADAP^-/-^ mice, including alveoli, bronchioles and bronchus of lungs (Fig 4C). This suggests that ADAP deficiency induces a possible link between reduced TGF-β1 production and declined lung function with acute lung inflammation.

### ADAP deficiency reduces TGF-1-induced CD103 expression in CD8^+^ T cells

**Fig 5:**
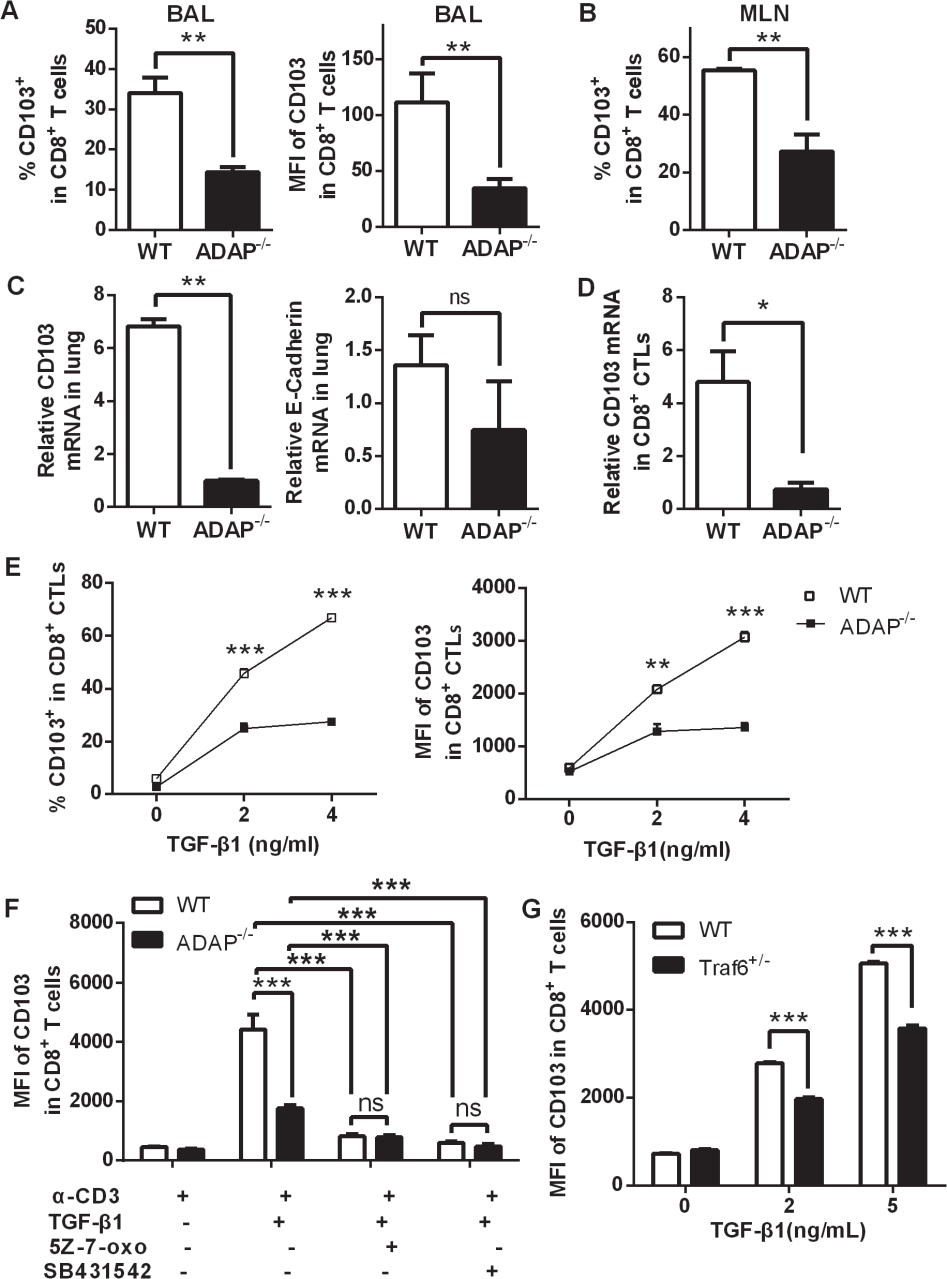
ADAP deficiency reduces TGF-β1-induced CD103 expression in CD8+ T cells. Wild type and ADAP^-/-^ mice were infected with the H5N1 virus (n = 3). At day 10 post infection, surface CD103 expression levels on CD8^+^ T cells from BAL or MLNs (A, B) or the mRNA levels of CD103 or E-cadherin of lungs (C) were measured. Data are representative of two independent experiments. (D) The mRNA levels of CD103 were examined in 10nM OVA_257-264_-stimulated CD8^+^ CTLs from wild type or ADAP^-/-^ OT1 Tg mice. Data are representative of two independent experiments. (E) Surface CD103 expression levels were examined from wild type or ADAP^-/-^ CD8^+^ T cells after treated with anti-CD3/CD28 (2ug/mL) and exogenous TGF-β1 (5ng/mL). Data are representative of two independent experiments. Data are representative of three independent experiments. (F) Surface levels of CD103 were examined from anti-CD3/CD28 and exogenous TGF-β1-stimulated wild type or ADAP^-/-^ CD8^+^ T cells, in the absence or presence of the TAK1 inhibitor (5Z-7-oxo; 2uM) or the TβRI inhibitor (SB431542; 10uM) respectively. Data are representative of two independent experiments. (G) Surface levels of CD103 were examined from wild type or TRAF6^+/-^ CD8^+^ T cells after stimulated with anti-CD3/28 antibody and exogenous TGF-β1. Data are representative of three independent experiments.

TGF-β1 signaling has been reported to induce and maintain integrin CD103 expression preferentially on influenza-specific CD8^+^ T cells, and CD103^+^CD8^+^ T cells plays a protective role in the control of influenza infection [13]. We therefore examined CD103 expression on ADAP deficient CD8^+^ T cells after H5N1 infection. Compared to that of the H5N1-infected wild type mice, surface CD103 expression on ADAP^-/-^ CD8^+^ T cells from BAL and MLNs was decreased (Fig 5A and 5B, percentages and MFI/Mean Fluorescence Intensity of CD103^+^ staining). The reduced CD103 mRNA levels were also observed in lungs of the infected ADAP^-/-^ mice (Fig 5C, left panel). The expression of the CD103 ligand E-cadherin was not significantly affected by ADAP deficiency, which is mainly expressed in lung endothelial cells (Fig 5C, right panel).

Next, we used exogenous TGF-β1 and anti-CD3/CD28 treatment to induce CD103 expression in wild type or ADAP deficient CD8^+^ CTLs *in vitro*. The mRNA levels of CD103 were profoundly reduced in ADAP^-/-^ CD8^+^ CTLs compared to those in wild type cells (Fig 5D). After treated with different concentrations of exogenous TGF-β1, surface CD103 expression was increased in wild type CD8^+^ T cells, while ADAP^-/-^ CD8^+^ T cells failed to significantly increase TGF-β1-induced CD103 expression (Fig 5E, percentages and MFI of CD103^+^ staining). We then asked whether ADAP co-operated with the TβRI-TRAF6-TAK1 pathway for TGF-β1-induced CD103 expression. The TAK1 inhibitors 5Z-7-oxozeaenol and the TβRI kinase inhibitor SB431542 significantly reduced surface CD103 expression in wild type CD8^+^ T cells, which reached to the same levels as that in the ADAP^-/-^ cells (Fig 5F). Since TRAF6^-/-^ mice are embryonic lethal, we isolated CD8^+^ T cells from TRAF6 heterozygous mice and the wild type control mice followed by stimulation with anti-CD3/CD28 in the presence of different concentrations of TGF-β1. Compared to wild type cells, TRAF6 heterozygous CD8^+^ T cells reduced surface CD103 expression by about 15–20% (Fig 5G). Together, we propose that TβRI, TAK1, TRAF6 and ADAP are indispensable for TGF-β1-induced CD103 expression.

### ADAP deficiency decreases VLA-1 expression in CD8^+^ T cells with increased amount of H5N1 virus

**Fig 6:**
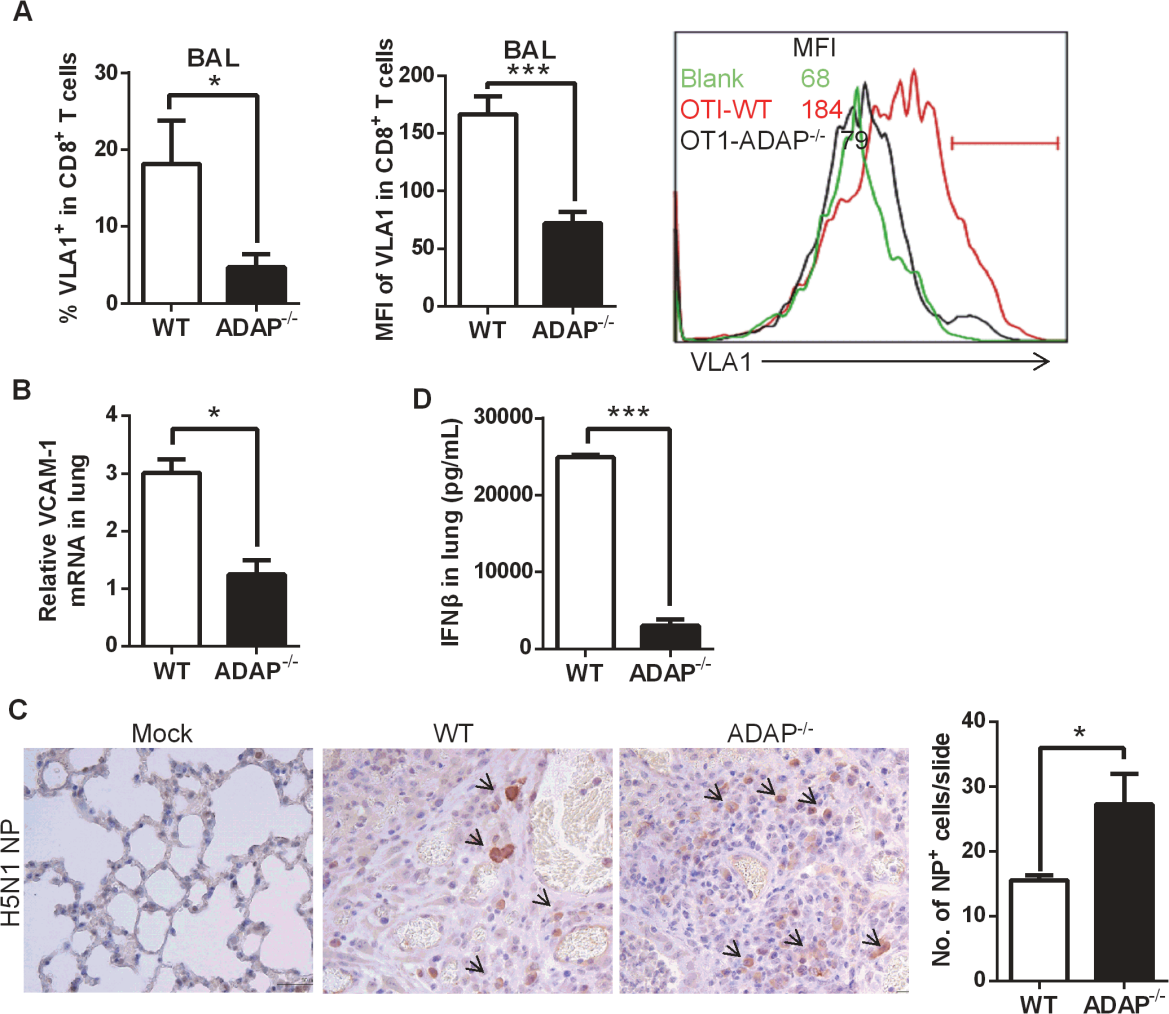
ADAP deficiency decreases VLA-1 expression in CD8+ T cells with increased amount of H5N1 virus. Wild type and ADAP^-/-^ mice were infected with the H5N1 virus for 10 days to measure the following parameters: surface VLA-1 levels in lung infiltrated CD8^+^ T cells (A); the mRNA levels of VCAM-1 in lungs from the infected mice (B); the amount of viral NP (nucleoprotein) in lungs by IHC staining (C); concentrations of IFN-β in lungs by ELISA (D).

As another critical integrin, VLA-1 (very late antigen-1, also termed α1β1) was reported to regulate CD8^+^ T cell-mediated immune protection against influenza infection [14]. In addition, β1 integrin has been suggested for LAP-TGF-β1 activation [35]. Interestingly, at day 10 post H5N1 virus infection, ADAP^-/-^ CD8^+^ CTLs in BAL reduced VLA-1 expression on cell surface (Fig 6A, percentages and MFI of VLA-1^+^ staining). The mRNA levels of the VLA-1 ligand VCAM-1 in the infected lungs from ADAP^-/-^ mice were also decreased (Fig 6B).

In agreement with the protective function of CD103 and VLA-1 in CD8^+^ T cells, wild type mice controlled H5N1 virus replication; while higher amount of H5N1 was detected in lungs of the infected ADAP^-/-^ mice at day 10 (Fig 6C, IHC staining with anti-H5N1 NP mAb). This was correlated with the reduced level of anti-viral cytokine IFN-β in lungs of the infected ADAP^-/-^ mice (Fig 6D). The copy numbers of H5N1 NP gene were measured by RT-PCR at different time points after infection. The amount of H5N1 virus reached the maximal level around day 4, then significantly declined at day 6 in lungs of H5N1-infected wild type mice. Although ADAP^-/-^ mice reduced the amount of H5N1 virus at day 6 compared to day 4, they were unable to clear virus effectively as wild type mice (S4 Fig).

### Rag1^-/-^ mice receiving ADAP^-/-^ T cells reduces protection and CD103 expression in response to H5N1 virus infection

**Fig 7:**
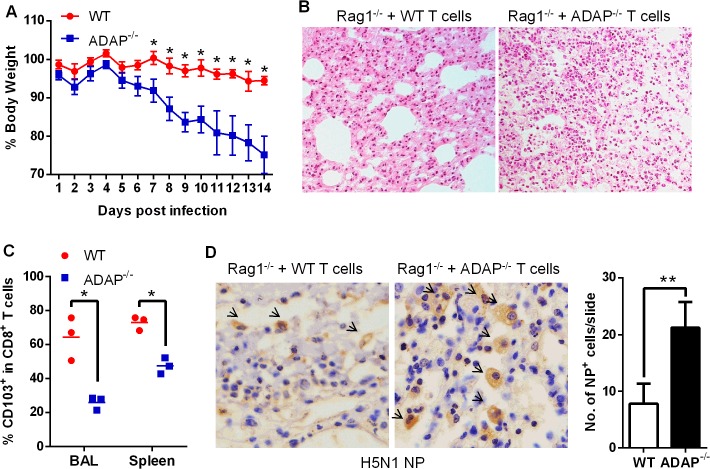
Rag1^-/-^ mice receiving ADAP^-/-^ T cells reduces protection in response to H5N1 virus infection. Four days after Rag1^-/-^ mice were transferred with 4×10^6^ CD4^+^ and 2.5×10^6^ CD8^+^ T cells purified from wild type or ADAP^-/-^ splenocytes, the recipient mice were then infected with the H5N1 virus GX/12 (10^6^ EID_50_) for the following analysis at day10 post infection (n = 5): bodyweight loss (A), HE staining of lungs (B), surface CD103 expression on the lung infiltrating CD8^+^ T cells (C), and the amount of viral NP (nucleoprotein) in lungs by IHC staining (D). Data are representative of two independent experiments.

To confirm that ADAP deficiency in T cells represented the main reason to cause more severe H5N1 virus infection, we transferred wild type or ADAP^-/-^ T cells into Rag1^-/-^ recipient mice. The comparable percentages of CD8^+^ T cells were detected in MLN and spleens in these Rag1^-/-^ mice, confirming a success of reconstitution of wild type or ADAP^-/-^ T cells (S5 Fig). Four days after adaptive T-cell transfer, these mice were infected with the H5N1 virus strain GX/12. Rag1^-/-^ mice received ADAP^-/-^ T cells significantly reduced body weight, compared to those receiving wild type T cells (Fig 7A). HE staining showed that more immune cells were infiltrated into lungs of the Rag1^-/-^ recipient mice that were adoptively transferred with ADAP^-/-^ T cells (Fig 7B). Furthermore, the lung infiltrating ADAP^-/-^ CD8^+^ T cells significantly reduced surface CD103 expression (Fig 7C). These findings have demonstrated that ADAP deficiency impairs T cell-mediated protection against H5N1 virus infection.

### ADAP^-/-^ mice enhance mortality with defective expression of TGF-1 and CD103 in response to H1N1 infection

**Fig 8:**
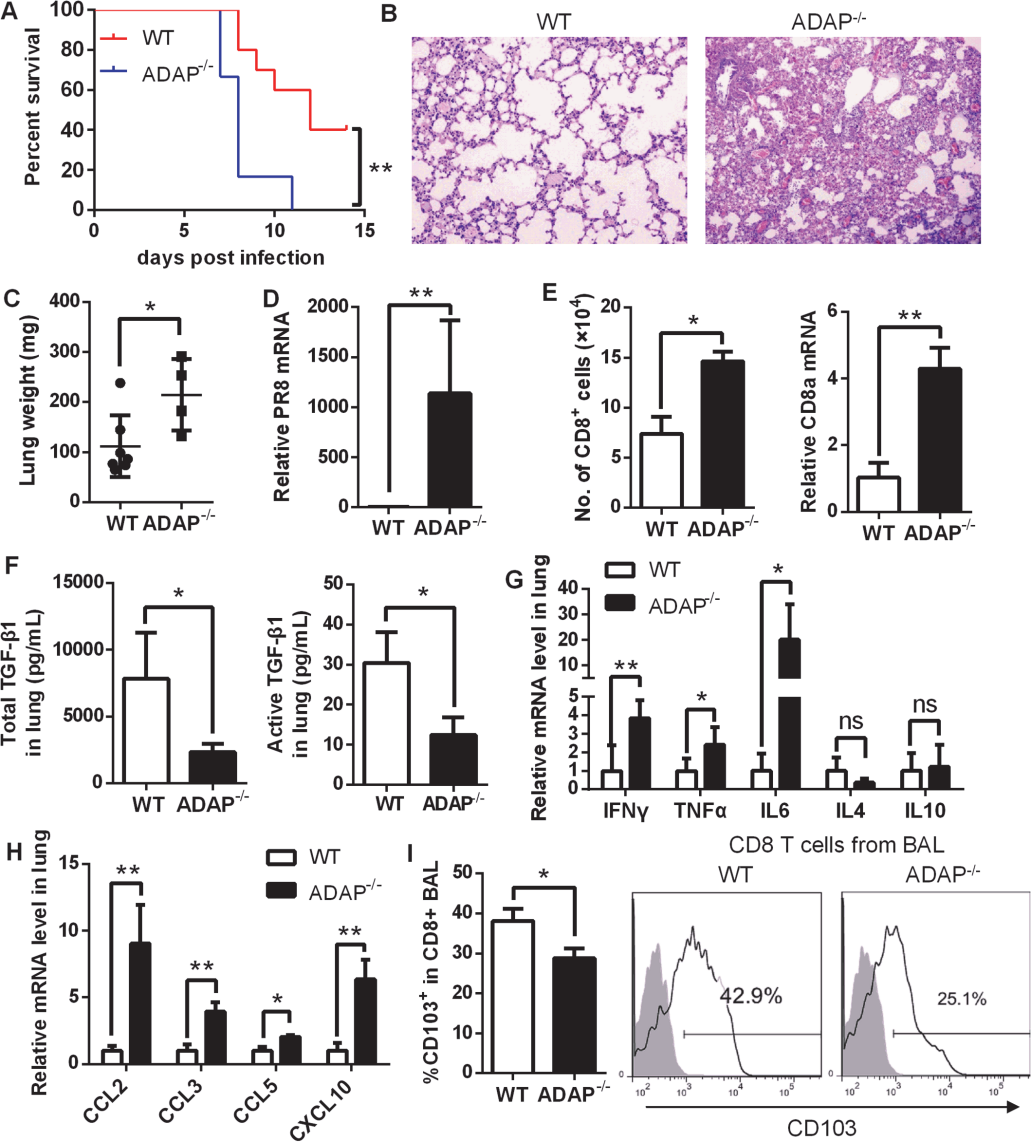
ADAP^-/-^ mice enhanced mortality with defective expression of TGF-β1 and CD103 in response to H1N1 infection. Wild type and ADAP^-/-^ mice were infected with 10 LD_50_ of A/PR8 to examine survival rates (A), HE staining of lungs (B), lung weight (C), the relative PR8 mRNA levels (D), amount of lung infiltrating CD8^+^ T cells (E), concentrations of total and active TGF-1 in lungs (F), the mRNA levels of the indicated cytokines (G) and chemokines (H), and surface CD103 levels on lung infiltrating CD8^+^ T cells (I) at day 8 post infection (WT: n = 7; ADAP^-/-^: n = 4). Data are representative of two independent experiments.

To assess whether ADAP regulates infection of other influenza virus subtype (i.e. not HPAI), wild type and ADAP^-/-^ mice were intranasally infected with the H1N1 virus strain A/PR8. Compared to wild type mice, ADAP^-/-^ mice showed heightened mortality within shorter periods after A/PR8 infection (Fig 8A). We observed that the A/PR8-infected ADAP^-/-^ mice had edematous lungs with enhanced weight and numbers of lung infiltrating immune cells (Fig 8B and 8C). At day 8 post infection, the mRNA level of viral PR8 in the lungs of ADAP deficient mice was significantly higher than that of wild type mice (Fig 8D). Moreover, the number of CD8^+^ T cells in BAL of lungs was significantly enhanced in the A/PR8-infected ADAP^-/-^ mice examined by FACS or real-time PCR (Fig 8E). Similar to the results of H5N1 infection, concentrations of active and total TGF-β1 in lungs were severely decreased in H1N1-infected ADAP^-/-^ mice compared to those from wild type mice (Fig 8F).

Previous studies have demonstrated that highly elevated IL-6, IL-1, TNF-α, IFN-γ, MIP, CXCL10 are major causes of ARDS (acute respiratory distress syndrome) and mortality [4, 6, 36, 37], and TGF-β1 could restrict inflammatory response. In agreement with the reduced TGF-β1 production, we detected markedly increased mRNA levels of IL-6, TNFα and IFNγ (Fig 8G) or CCL2, CCL3, CCL5 and CXCL10 (Fig 8H) in lungs from A/PR8-infected ADAP^-/-^ mice. By contrast, IL-4 or IL10 expression was not substantially changed (Fig 8G). Similar to H5N1 infection, we also detected reduced surface CD103 levels in lung infiltrating ADAP deficient CD8^+^ T cells (Fig 8I). Taken together, ADAP enhances TGF-β1 production and signaling, and is indispensable for protection against H1N1 and H5N1 virus infection.

## Discussion

As the highly pathogenic avian influenza (HPAI), H5N1 virus infection induces unbalanced host immune responses with highly elevated cytokines and significant lung pathology [36–38]. TGF-β1 is suggested to suppress cytokine storm during H5N1 virus infection [10]. Since HPAI loads and replicates in the respiratory tract for much longer time than seasonal influenza [2], viral antigen activated T cells become one of the major sources to produce TGF-β1. In this study, we observed the enhanced mortality in the H5N1- or H1N1-infected ADAP KO mice and this could be possibly explained by three main findings including (1) defective TGF-β production and TGF-β signaling; (2) cytokine storm; (3) reduced CD103 expression. Previous studies suggest the protective role of TGF-β to control initiation and resolution of inflammation, while CD103^+^CD8^+^ T cell-mediated immune responses play vital roles in recovery from H5N1 virus infection [39]. Our study has provided the first evidence that CD8^+^ T cells secreted TGF-β1 in an autocrine manner via the TβRI-TRAF6/ADAP/TAK1-SMAD3 pathway (Fig 1). Further, ADAP forms a complex with TRAF6/TAK1 in CD8^+^ T cells to regulate SMAD3 activation and TGF-β-induced CD103 expression (Figs 2 and 4). Consistent with the *in vitro* findings, H5N1-infected ADAP KO mice reduce TGF-β production and CD103 expression (Figs 3 and 5). Moreover, the reduced TGF-β production was correlated to the elevated levels of inflammatory cytokines and chemokines in H5N1- or H1N1-infected ADAP KO mice (Figs 3 and 8). We further used Rag1 KO mice that were adoptively transferred with ADAP KO T cells to demonstrate that ADAP deficient T cells play a direct role in protection against virus infection (Fig 7). These observations together suggest the enhanced mortality in ADAP KO mice was closely related to the defective TGF-β production and signaling. To demonstrate a direct link of the ADAP KO mortality phenotype to TGF-β, it is better to set a ‘rescue’ experiment provided by TGF-β1 or TGF-β1 receptor, which protects ADAP KO mice effectively as the wild type control mice. Since ADAP deficient T cells not only decreased TGF-β1 production, but also poorly responded to TGF-β1 signaling, we were unable to expect a miraculous reversal of lung pathology or reduction of mortality in ADAP^-/-^ mice after exogenous injection of TGF-β1. Together, this study suggests that ADAP is indispensable for TGF-β1 production and TGF-β1-triggered CD103 expression via the TβRI-, TRAF6-, TAK1-dependent manner and protects from influenza virus infection.

Although mature TGF-β1 plays a critical role in the suppression of cytokine storm, H5N1 virus fails to activate the latent form of TGF-β1 (LAP-TGF-β1) [10]. We have observed that ADAP controls surface expression levels of VLA-1 (i.e. α1β1) in lung infiltrating CD8^+^ T cells during H5N1 virus infection (Fig 6). β1 integrin has been suggested for LAP-TGF-β1 activation [35], and TGF-β1 regulates expression of various β1 integrins. It is likely that ADAP is indispensable for CD8^+^ T cells to (1) promote VLA-1 expression, which activates LAP-TGF-β1 on neighboring immune cells to release active TGF-β1; (2) produce autocrine or paracrine TGF-β1, which then binds TβRI on CD8^+^ T cells to amplify the reciprocal TGF-β1-integrin crosstalk.

Interestingly, the combination of TGF-β1 and TCR stimulation could induce CD103 expression as well as FoxP3 expression, which are both dependent on SMAD2/3 and NFAT1 [40]. In agreement with this line, alloantigen-induced CD103^+^CD8^+^ T cells display strong suppressive activity in a mixed lymphocyte culture [41]. In addition, our recent findings suggest that ADAP deficient CD8^+^ T cells enhance cytotoxicity to kill tumor cells. Since ADAP deficient CD8^+^ T cells reduce TGF-β1/CD103 expression and decrease the ability to protect from H5N1 virus infection, further investigation should ask whether ADAP regulates suppressive function of CD103^+^CD8^+^ T cells, whether the TGF-β1/TβRI-ADAP-TRAF6-TAK1 pathway represents a general mechanism for reciprocal TGF-β1-integrin crosstalk to mediate inflammation-associated autoimmune diseases or graft transplantation.

Past efforts have identified various SNPs (Single Nucleotide Polymorphisms) or mutations in key molecules of TGF-β1 pathway, which are associated with inflammatory, infections or human cancer including *TGFB1*, *TβRI/II*, *SMADs*, *Foxo3A* [42–44]. Interestingly, a recent study has found an association between polymorphisms located in *ADAP/FYB* gene and Systemic Lupus Erythematosus (SLE) [45]. Considering high mortality of humans in H5N1 virus infection, it is critical to investigate whether these human SNPs or mutations in TGF-β1-regulated pathway (including the *ADAP* gene) could possibly enhance disease susceptibility or clinical manifestations in response to acute H5N1 virus infection.

## Methods

### Mice and ethics statement

ADAP^-/-^ mice and OT-1 TCR transgenic mice were kindly provided by Drs. E. Peterson (University of Minnesota, USA) and CE Rudd (University of Cambridge, UK) respectively. ADAP^-/-^ mice were crossed with OT1 TCR transgenic mice to generate ADAP^-/-^ OT1 mice. RAG1^-/-^ mice and wild type C57BL/6 mice were respectively purchased from Model Animal Research Center of Nanjing University or Shanghai Slac Laboratory Animal co. Ltd. Mice were bred under specific pathogen-free conditions at the Animal Care Facility of Shanghai Institute of Biochemistry and Cell Biology, Chinese Academy of Sciences. Sex and age-matched mice (8–12 weeks old) were used and animal infection with the H5N1 avian influenza virus A/Duck/Guangxi/12/2003 (GX12) were conducted in BSL3+ approved by the Chinese Ministry of Agriculture. This study was carried out in strict accordance with the recommendations in the Guide for the Care and Use of Laboratory Animals of the Ministry of Science and Technology of the People's Republic of China. The protocols for animal studies were approved by the Committee on the Ethics of Animal Experiments of the Harbin Veterinary Research Institute (HVRI) of the Chinese Academy of Agricultural Sciences (CAAS) (approval numbers BRDW-XBS–12).

### Viral infection

The H5N1 virus A/Duck/Guangxi/12/2003 (termed GX/12) was used which was isolated from ducks in China. The viruses were propagated in 10-day-old specific-pathogen-free embryonated eggs and were stored at -70°C. Mice were lightly anesthetized with CO_2_ and were challenged intranasally with 10^6^ 50% egg infectious doses (EID_50_) of GX/12 in a volume of 50 μl. A/PR8 virus, kindly provided by Prof. Dongming Zhou (Institut Pasteur of Shanghai, Chinese Academy of Sciences, Shanghai, China), was grown in embryonated chicken eggs, and titrated for mean lethal dose (LD50) in adult C57BL/6 mice. C57BL/6 mice were inoculated intranasally with 10 LD50 of A/PR8 virus. At the indicated time points, samples from the infected mice were collected for RNA extraction or preparation of tissue lymphocytes. The remaining mice were monitored for 14 days for weight loss and mortality. Animal experiments were performed at least two independent times.

### T cell adoptive transfer

Splenocytes were collected from ADAP^-/-^ or wild type control mice, then CD4^+^ and CD8^+^ T cells were purified by positive selection with BD IMag™ CD4^+^ and CD8^+^ Magnetic Particles respectively. 4×10^6^ CD4^+^ and 2.5×10^6^ CD8^+^ T cells were transferred into Rag1^-/-^ mice by tail vein injection. After 4 days, the recipient mice were challenged with the H5N1 virus (GX/12).

### Preparation of tissue lymphocytes

Briefly, BAL fluids from the infected mice were collected by lavage with 1ml PBS for three to five times. Or, MLN and spleen were homogenized, filtered through 70 μm nylon mesh; and red blood cell were lysed and removed. Immune cells were collected after extensive wash with PBS.

### ELISA assay

Lung was harvested and homogenized in 1mL 1×PBS on ice. Serum was gained in blood after clots were formed at 4°C. TGF-β1 concentrations in lung homogenate, serum, and supernatant from cell cultures were quantified by ELISA according to the manufacture’s instruction (Westong Biotechnology, Shanghai). The TGF-β ELISA kit recognizes the mature/active form of TGF-β1 and does not detect the latent form of TGF-β1. To measure the total concentration of TGF-β1 (both active and latent form), samples required acid-treatment and neutralization to remove LAP from the latent TGF-β1. Active TGF-β1 in samples was assayed without acid treatment.

### Histology and immunohistochemistry

Lungs from the H5N1 virus infected mice were collected at the indicated time points, fixed in formalin and embedded in paraffin. Sections were cut and stained with hematoxylin and eosin (H&E) or immunohistochemistry (IHC). The relative degree of lung inflammation and tissue damage were examined and scored using the method described previously [46, 47]. Briefly, lung infiltration of inflammatory cells was scored as follows: 0, no inflammation; 1, mild peribronchial and peribronchiolar infiltrates, extending throughout, 10% of the lung; 2, moderate inflammation covering 10–50% of the lung; 3, severe inflammation involving over one-half of the lung. Degeneration of bronchi and bronchiolar epithelium, alveoli degeneration and collapse was scored as follows: 0, no degeneration; 1, little vacuolar degeneration of bronchi and bronchial epithelium cells, normal pulmonary alveoli; 2, mild necrosis of bronchi and bronchial epithelium, mild alveoli damage; 3, severe degeneration. Necrosis was scored as follows: 0, no necrosis; 1, mild necrosis with scant exudate; 2, marked necrosis with abundant exudate; 3, severe interstitial edema around blood vessels, apparent injured parenchyma and alveolar epithelial cells with greater infiltration of inflammatory cells.

### TGF-β1 induction *in vitro*

To check whether ADAP regulated TGF-β1 production *in vitro*, naïve splenocytes from WT or ADAP^-/-^ OT1 Tg mice were stimulated with 10nM OVA_257-264_ and 20U/ml human recombinant IL-2 for 3 days to generate CD8^+^ CTLs. Alternatively, splenocytes from WT or ADAP^-/-^ mice or C8166 T cell lines overexpressing GFP or ADAP-GFP were stimulated with 2ug/mL plate bound anti-CD3/CD28 antibody for 3 days. Cells were harvested to examine surface expression by anti-LAP (TGF-β1) staining or TGF-β1 mRNA levels by RT-PCR. To block TGF-β signaling, splenocytes from WT or ADAP^-/-^ OT1 Tg mice were stimulated with 10nM OVA_257-264_ and 5ng/mL exogenous TGF-β1 in presence or absence of the TGF-β receptor I kinase inhibitor SD208 or SB431542 (10uM). TGF-β1 mRNA levels were then measured by RT-PCR.

### Flow cytometry

Lymphocytes from each organ was stained with various combinations of mAbs to CD4-FITC/APC, CD8α-PE/APC, CD3α-PE, and B220-FITC (eBiosciences), CD103-APC, LAP (TGF-β1)-PE (Clone TW7-20B9, Biolegend). Fixation/Permeabilization Concentrate and Diluent Kit (eBiosciences) were used for the intracellular staining of FoxP3-PE/APC according to the user’s handbook. After stained with surface markers, cells were stained with Annexin V and PI (BD Pharmingen) to examine apoptosis. For *in vivo* BrdU incorporation assay, mice were administered of BrdU (16 mg/ml; 50 μl) via i.n. route [48]; cells were harvested after 24hours and stained with BD FastImmune BrdU kit (BD Biosciences). Samples were run on BD Biosciences FACSCalibur, FACSAria or Accuri C6 instruments, and analyzed with FlowJo software (Tree Star, Ashland, OR).

### Luciferase reporter assay

Wild type Jurkat, ADAP deficient Jurkat (JDAP cells) or ADAP overexpressing Jurkat cells were transfected with (CAGA)_12_-luciferase or (SBE)_4_-luciferase reporter plasmids respectively. Cells were then stimulated with 5ng/mL exogenous TGF-β1 for 6–8 hrs, followed by measurement of luciferase activity using Dual-Glo luciferase system (Gibco).

### Western blot assay

CD8^+^ CTL cells generated with 10nM OVA_257-264_ treatment from WT and ADAP^-/-^ OT1 Tg mice were left unstimulated or stimulated with 5ug/mL TGF-β1 for different time points. Cell lysates were prepared for immunoblotting with the indicated antibodies. To quantify the amount of nuclear SMAD3, WT and ADAP KO CD8^+^ CTLs were treated with 5ng/mL TGF-β1 for 30min. The extraction of cytoplasmic and nuclear protein was performed according to the product manufacturer instructions. Equal amounts of cytoplasmic and nuclear extract were loaded for western blotting assay with antibodies against SMAD3, SP1 and tubulin.

### RNA extraction and real-time PCR analysis

Total RNA was extracted from tissues or cells using Trizol reagent (Invitrogen) following the supplier’s protocol and first-strand cDNA was synthesized using M-MLV Reverse Transcriptase RNase H Minus (Promega). Relative quantitative Real-Time PCR was carried out on BIO-RAD CFX96 Touch ^TM^ Real-Time PCR Detection System (BIO RAD) using Bestar ^TM^ Real-Time PCR Master Mix (SYBR Green methods, DBI Bioscience) with 2^-△△^CT method. Actin was used as the reference gene. All primer sequences are listed in Table 1.

**Table 1:** Primers used for RT-PCR.

β-actin Forward	5’-CGTTGACATCCGTAAAGACC
β-actin Reverse	5’-TAGGAGCCAGAGCAGTAATC
CCL-2 Forward	5’-CCAGCAAGATGATCCCAATG
CCL-2 Reverse	5’-TGGTTCCGATCCAGGTTTT
CCL-3 Forward	5’-CCCAGCCAGGTGTCATTT
CCL-3 Reverse	5’-GCATTCAGTTCCAGGTCAGT
CCL-5 Forward	5’-GACACCACTCCCTGCTGCTT
CCL-5 Reverse	5’-CACTTGGCGGTTCCTTCG
CD103 Forward	5’-GCCGTGATCCAGACTGAGTTTGAT
CD103 Reverse	5’-ATGGCTGAGGCGGTCTTAGTGACT
CXCL-10 Forward	5’-CATCCCGAGCCAACCTTC
CXCL-10 Reverse	5’-GCTTCCCTATGGCCCTCA
E-cadherin Forward	5’-TGGAGGAATTCTTGCTTTGC
E-cadherin Reverse	5’-CGTACATGTCAGCCAGCTTC
IFN-γ Forward	5’-ATGAACGCTACACACTGCATC
IFN-γ Reverse	5’-CCATCCTTTTGCCAGTTCCTC
IL-1β Forward	5’-CTGGTACATCAGCACCTCAC
IL-1β Reverse	5’-AGAAACAGTCCAGCCCATAC
IL-4 Forward	5’-GGTCTCAACCCCCAGCTAGT
IL-4 Reverse	5’-GCCGATGATCTCTCTCAAGTGAT
IL-6 Forward	5’-TGTATGAACAACGATGATGCACTT
IL-6 Reverse	5’-ACTCTGGCTTTGTCTTTCTTGTTATCT
NP(H5N1) Forward	5’-GGGTCCCACAGCAACGATG
NP(H5N1) Reverse	5’-CACCAGGGAATGCACGTCC
TGF-β1 Forward	5’-CACTGATACGCCTGAGTG
TGF-β1 Reverse	5’-CTCCCGTGGCTTCTAGTGC
TNF-α Forward	5’-AGTGACAAGCCTGTAGCCC
TNF-α Reverse	5’-GAGGTTGACTTTCTCCTGGTAT
VLA-1 Forward	5’-CCTGTACTGTACCCAATTGGATGG
VLA-1 Reverse	5’-GTGCTCTTATGAAAGTCGGTTTCC

### CD103 induction *in vitro*

Naïve splenocytes from WT and ADAP^-/-^ OT1 Tg mice were stimulated with 10nM OVA_257-264_ in presence of different concentrations of TGF-β1 for 3 days, CD103^+^CD8^+^ CTLs were identified by staining with anti-mouse CD8-FITC and anti-mouse CD103-APC. Alternatively, to examine surface CD103 expression, CD8^+^ T cells were purified with BD IMag™ CD8+ Magnetic Particles; WT and ADAP^-/-^ CD8^+^ T cells were stimulated with 2ug/mL plate bound anti-CD3/CD28 and 5ng/mL exogenous TGF-β1 in the presence of the TAK1 inhibitor 5Z-7-oxo (2uM) or the TGF-β receptor I kinase inhibitor SB431542 (10uM); WT and TRAF6^+/-^ CD8^+^ T cells were stimulated with 2ug/mL plate bound anti-CD3/CD28 and different concentrations of exogenous TGF-β1 for 3 days.

### Statistical analysis

Data were analyzed using unpaired two tailed Student’s *t* test. Survival rates were analyzed by log-rank (Mantel-Cox) test. *p* value <0.05 or <0.01 was marked as * or ** respectively.

## Supporting Information

S1 Fig (A) ADAP was overexpressed with Myc-tagged SMAD2 or HA-tagged SMAD3 in 293T cells, followed by immunoprecipitation using anti-Myc or anti-HA antibodies and immunoblotting with the indicated antibodies. (B) Wild type or ADAP^-/-^ CD8^+^ OT1 Tg cells were stimulated with 10nM OVA_257-264_ peptide and 5ng/mL exogenous TGF-β1 as the indicated time points. The relative ratio between p-p38, p-JNK, p-p85 (Tyr458) or p-AKT (Ser473) and β-actin was measured. (C) The interaction between EGFP-ADAP and Flag-TRAF6 or HA-TAK1 was examined by IPs in 293T cells after transfected with the indicated plasmids. Data are representative of two independent experiments.
(TIF)

S2 Fig (A) The numbers of lung infiltrating CD8^+^ T cells from wild type or ADAP^-/-^ mice were checked at different days after H5N1 virus infection. (B) The percentages of lung infiltrating regulatory T cells were examined by FACS staining with anti-CD4 and anti-Foxp3 antibodies at day 10 post infection.
(TIF)

S3 Fig (A, B and C) The BrdU incorporation assay and PI/Annexin V staining of CD8^+^ T cells from lungs, MLN, spleens of H5N1-infected ADAP^-/-^ and wild type mice. (D) The total number of CD8^+^ T cells from spleens of H5N1-infected ADAP^-/-^ and wild type mice.
(TIF)

S4 Fig The copy numbers of the H5N1 strain GX/12 in lungs of ADAP^-/-^ and wild type mice were measured by RT-PCR at different days post infection. (TIF)

S5 Fig Related to Fig 7. At day 10 post GX12 infection, the percentages of CD8^+^ T cells were checked in BAL, MLN and spleens in these reconstituted Rag1^-/-^ mice.
(TIF)
