# Supplementary figures and images for: The Immune Adaptor ADAP Regulates Reciprocal TGF-β1-Integrin Crosstalk to Protect from Influenza Virus Infection
Source: PLoS Pathog. 2015 Apr 24;11(4):e1004824. doi: 10.1371/journal.ppat.1004824 (PMC4409120; doi:10.1371/journal.ppat.1004824)

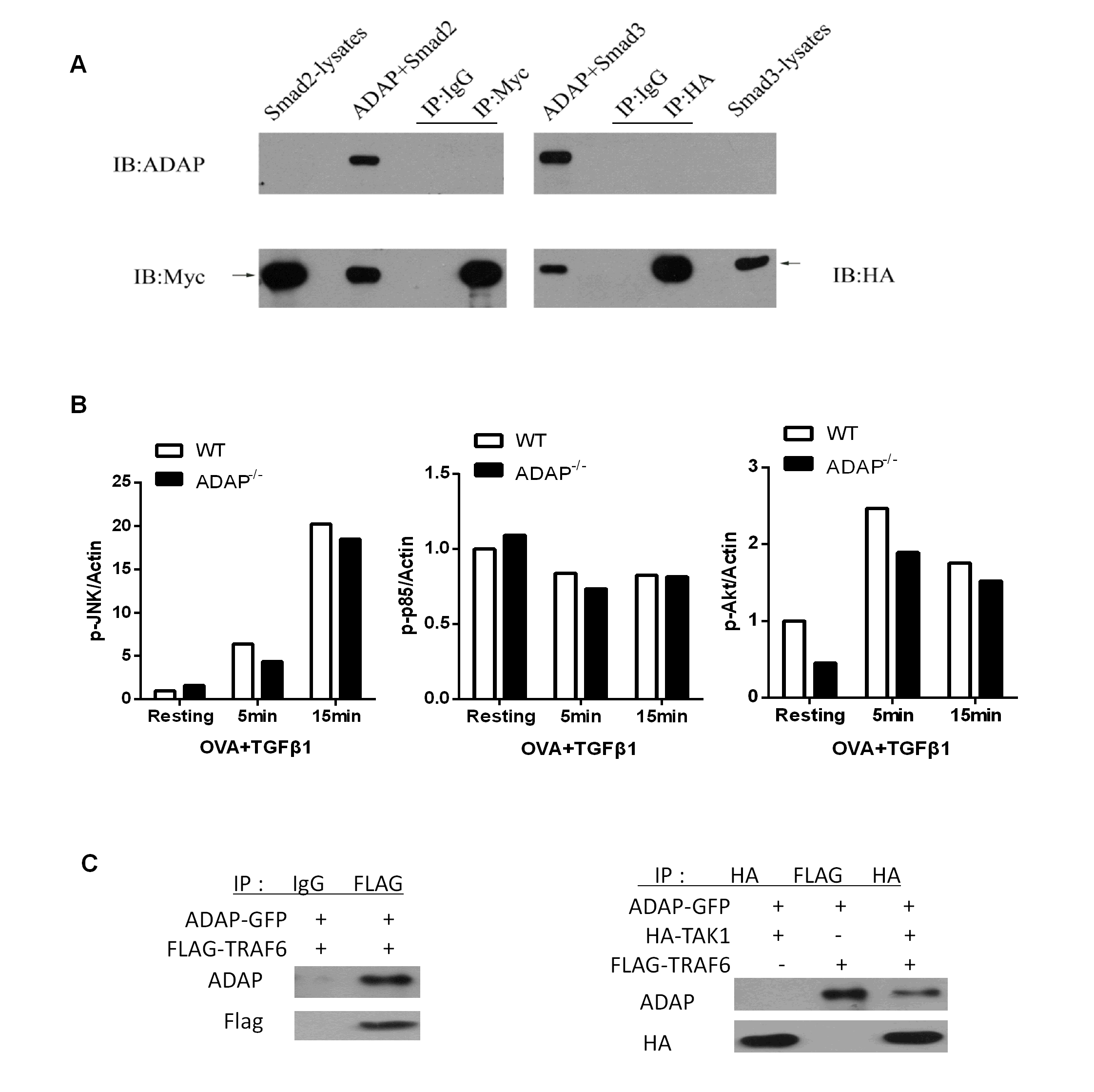

Supplement: S1 Fig — (B) Wild type or ADAP-/- CD8+ OT1 Tg cells were stimulated with 10nM OVA257-264 peptide and 5ng/mL exogenous TGF-β1 as the indicated time points. The relative ratio between p-p38, p-JNK, p-p85 (Tyr458) or p-AKT (Ser473) and β-actin was measured. (C) The interaction between EGFP-ADAP and Flag-TRAF6 or HA-TAK1 was examined by IPs in 293T cells after transfected with the indicated plasmids. Data are representative of two independent experiments. (TIF) [file ppat.1004824.s001.tif]

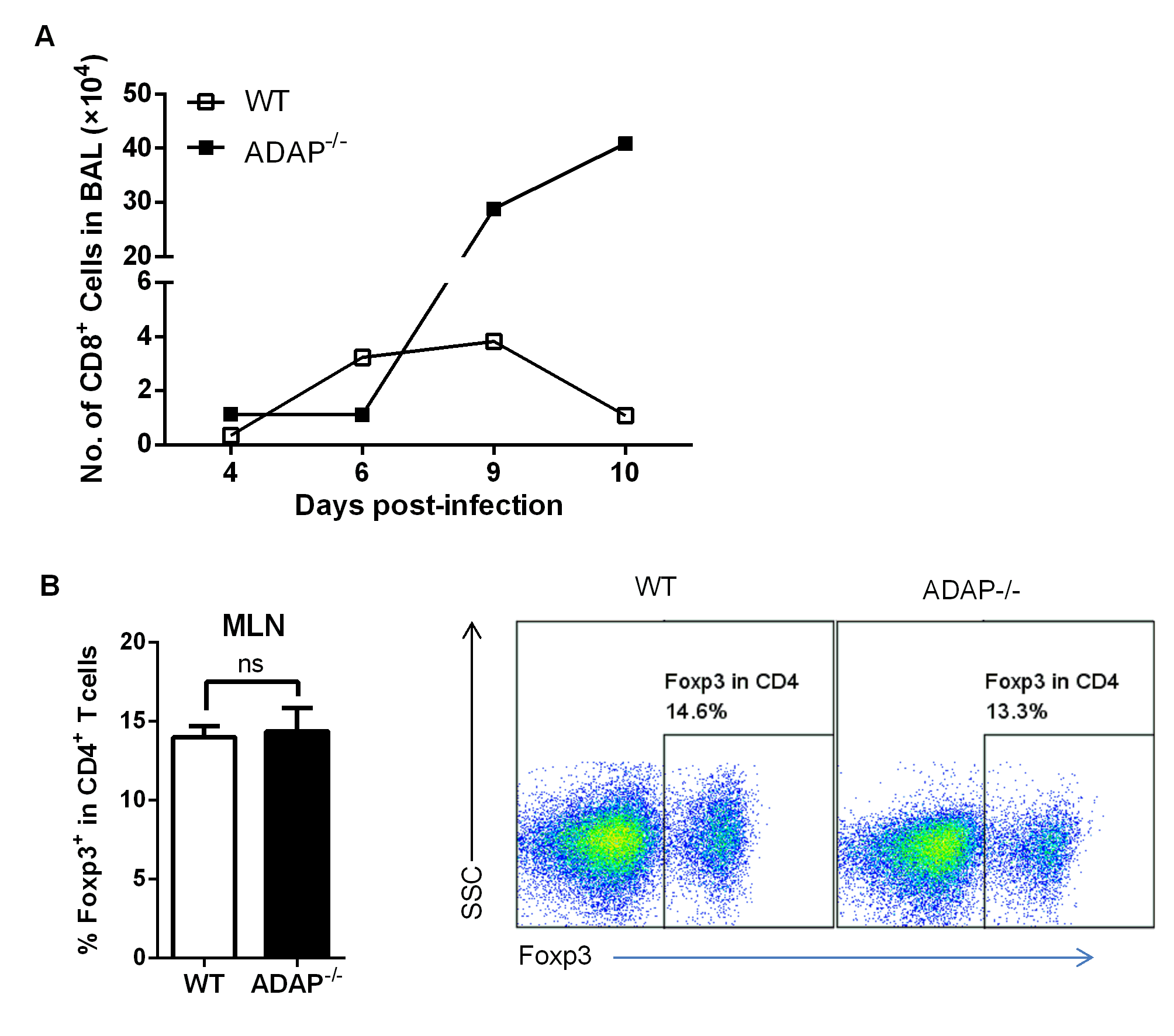

Supplement: S2 Fig — (B) The percentages of lung infiltrating regulatory T cells were examined by FACS staining with anti-CD4 and anti-Foxp3 antibodies at day 10 post infection. (TIF) [file ppat.1004824.s002.tif]

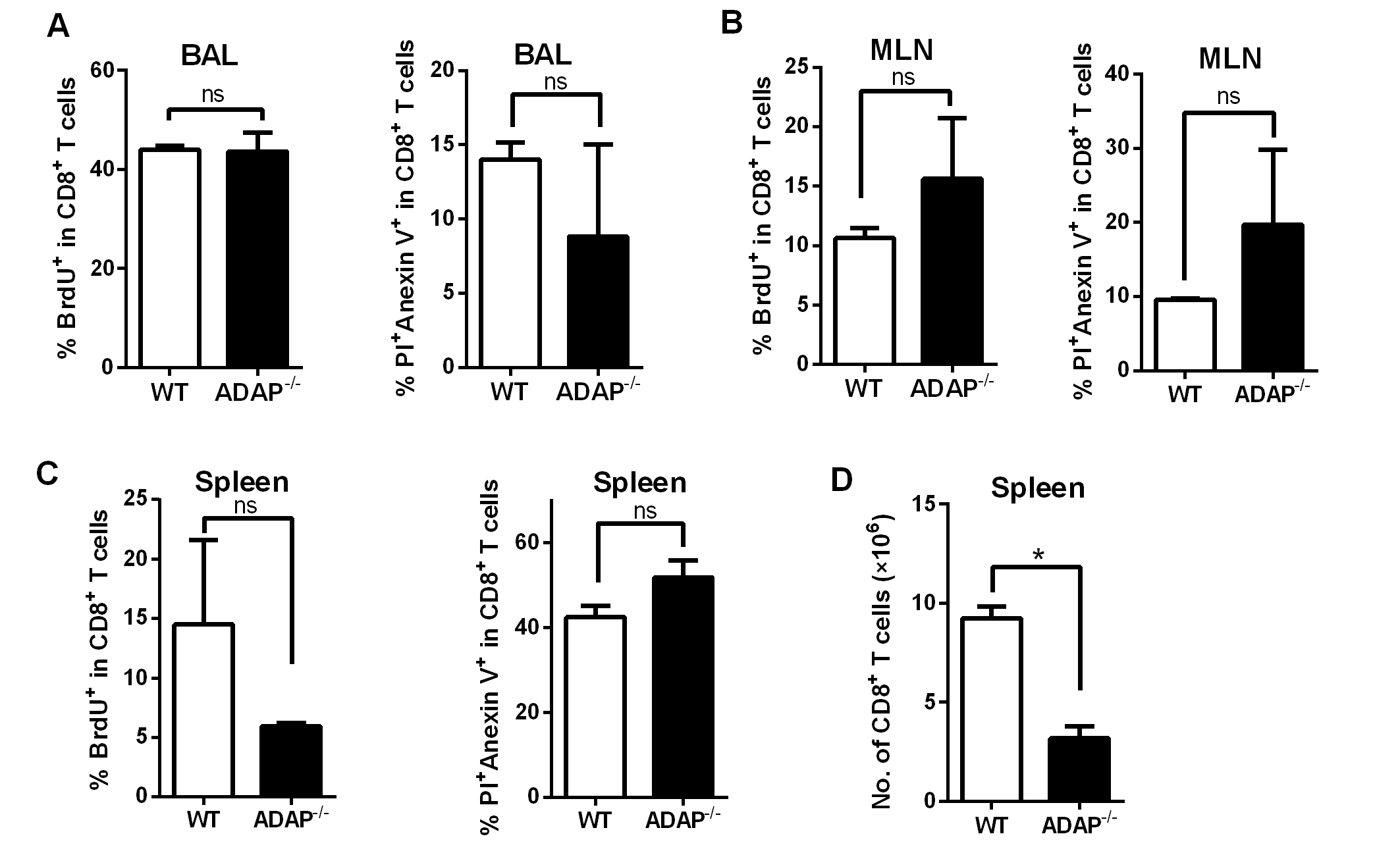

Supplement: S3 Fig — (D) The total number of CD8+ T cells from spleens of H5N1-infected ADAP-/- and wild type mice. (TIF) [file ppat.1004824.s003.tif]

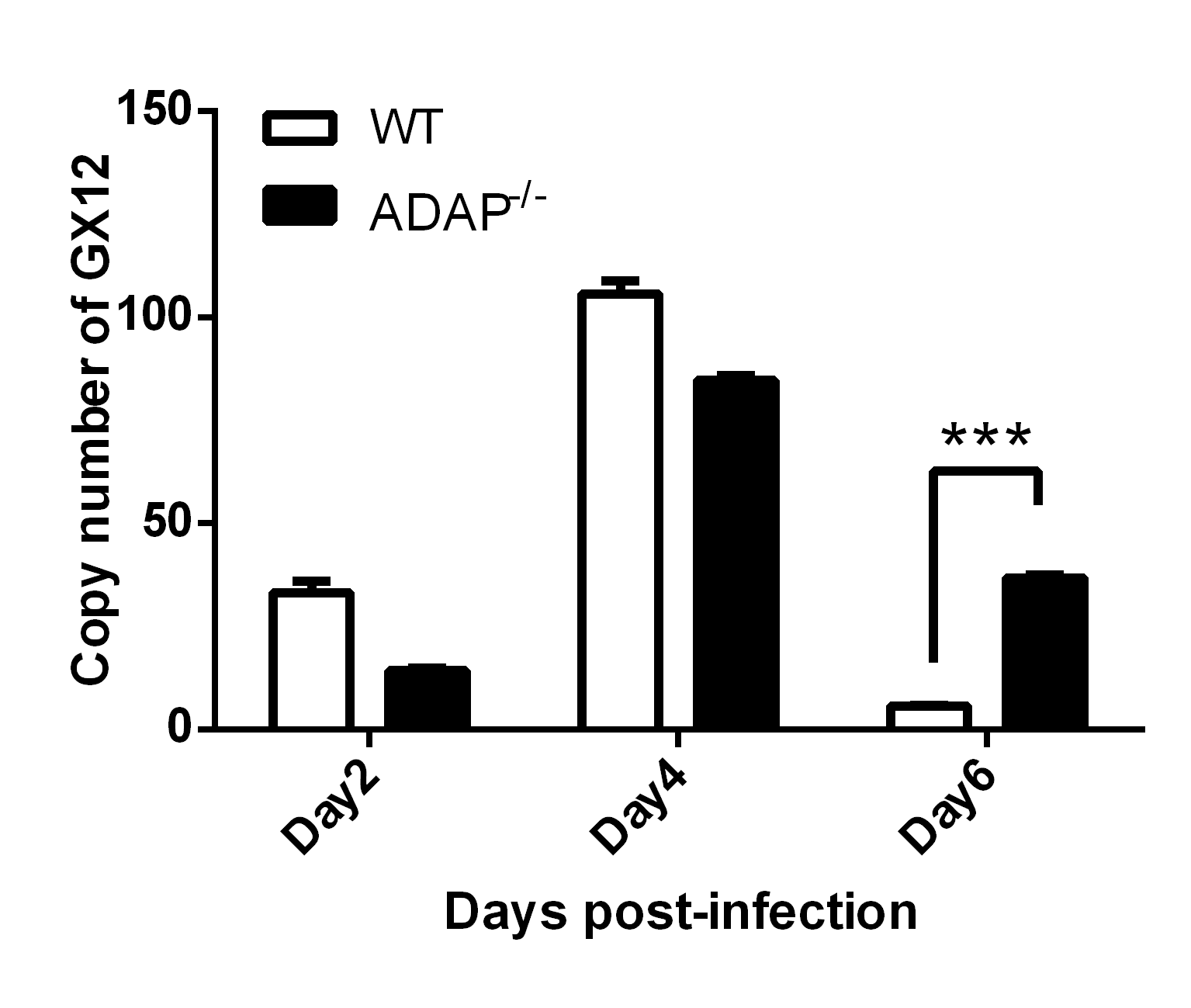

Supplement: S4 Fig — (TIF) [file ppat.1004824.s004.tif]

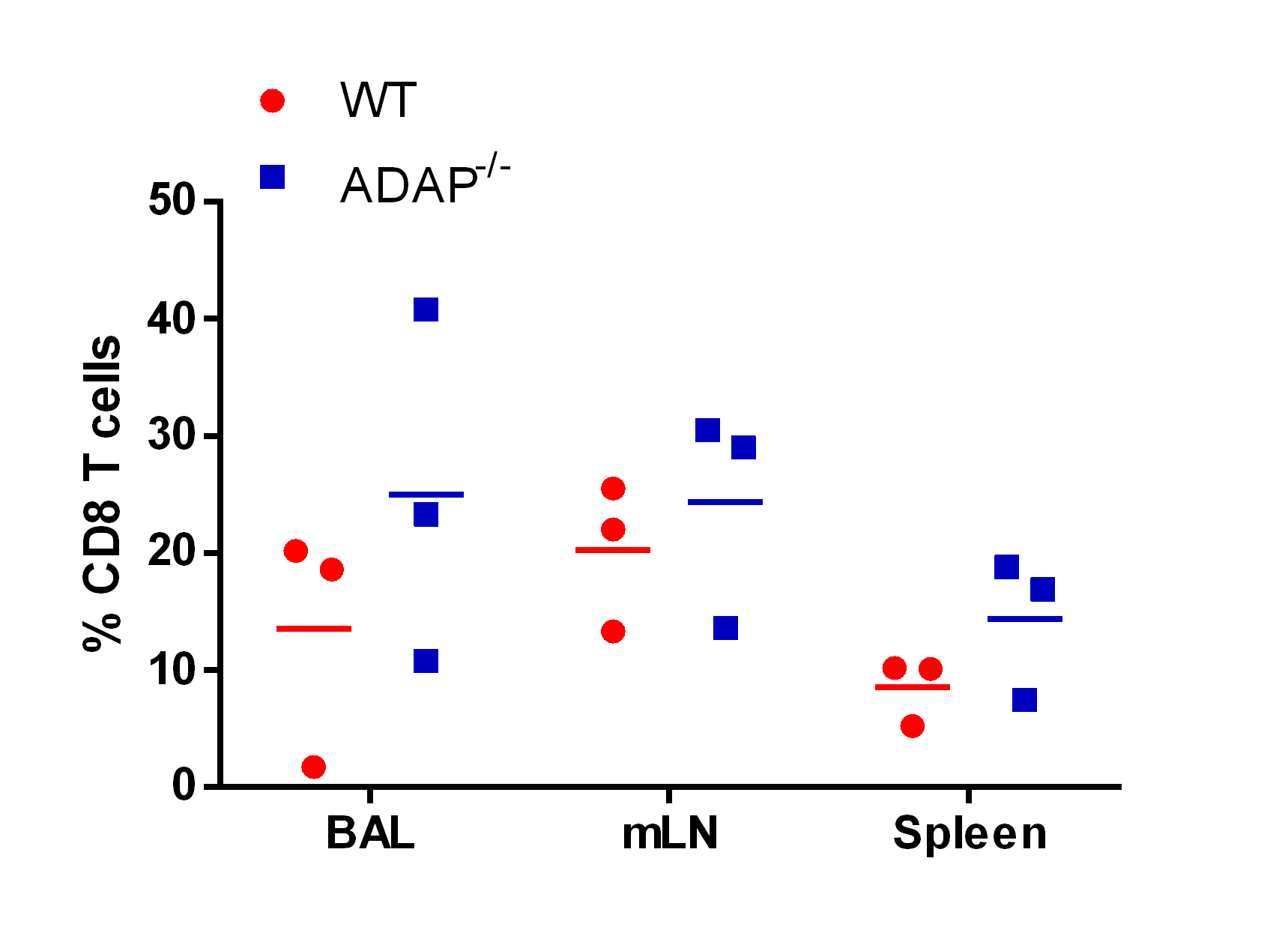

Supplement: S5 Fig — At day 10 post GX12 infection, the percentages of CD8+ T cells were checked in BAL, MLN and spleens in these reconstituted Rag1-/- mice. (TIF) [file ppat.1004824.s005.tif]
